# Role of Active Site Rigidity in Activity: MD Simulation and Fluorescence Study on a Lipase Mutant

**DOI:** 10.1371/journal.pone.0035188

**Published:** 2012-04-13

**Authors:** Md. Zahid Kamal, Tabrez Anwar Shamim Mohammad, G. Krishnamoorthy, Nalam Madhusudhana Rao

**Affiliations:** 1 Centre for Cellular and Molecular Biology (Council for Scientific and Industrial Research), Hyderabad, India; 2 Centre for DNA Fingerprinting and Diagnostics, Nampally, Hyderabad, India; 3 Department of Chemical Sciences, Tata Institute for Fundamental Research, Mumbai, India; University of South Florida, United States of America

## Abstract

Relationship between stability and activity of enzymes is maintained by underlying conformational flexibility. In thermophilic enzymes, a decrease in flexibility causes low enzyme activity while in less stable proteins such as mesophiles and psychrophiles, an increase in flexibility is associated with enhanced enzyme activity. Recently, we identified a mutant of a lipase whose stability and activity were enhanced simultaneously. In this work, we probed the conformational dynamics of the mutant and the wild type lipase, particularly flexibility of their active site using molecular dynamic simulations and time-resolved fluorescence techniques. In contrast to the earlier observations, our data show that active site of the mutant is more rigid than wild type enzyme. Further investigation suggests that this lipase needs minimal reorganization/flexibility of active site residues during its catalytic cycle. Molecular dynamic simulations suggest that catalytically competent active site geometry of the mutant is relatively more preserved than wild type lipase, which might have led to its higher enzyme activity. Our study implies that widely accepted positive correlation between conformation flexibility and enzyme activity need not be stringent and draws attention to the possibility that high enzyme activity can still be accomplished in a rigid active site and stable protein structures. This finding has a significant implication towards better understanding of involvement of dynamic motions in enzyme catalysis and enzyme engineering through mutations in active site.

## Introduction

Understanding the relationship of enzyme conformational flexibility with its stability and activity is a very active area of research. Many studies comparing conformational flexibility of homologous thermophilic-mesophilic enzyme pairs, using various techniques like fluorescence quenching [1], molecular dynamics simulation [2], hydrogen/deuterium exchanges [3], [4] and NMR [5], have shown that conformational flexibility in thermophilic enzymes at room temperature is lower than mesophilic enzymes. It has been inferred that reduced conformational flexibility of thermophilic proteins is a direct consequence of conformational stabilization and vice versa. Interestingly, activity of thermophilic enzymes is also lower than mesophilic homologues [3], [6] leading to belief that lower flexibility in thermophilic enzymes is insufficient in supporting the necessary motions required by enzymes for catalysis. This belief is further supported by the finding that thermophilic enzymes often showed comparable conformational flexibility as well as activity to their mesophilic homologues at their respective habitat temperatures [3], [5]. These led to the view point that increase in protein stability is always associated with decrease in conformation flexibility which in turn leads to reduction in enzyme activity. However, in recent years many thermostable enzymes have been identified in various laboratories, which are both more stable and have comparable or even higher activity (∼1–7 fold) at lower temperatures than their parents [7]–[13]. These evidences argue that high activity and high stability are not mutually exclusive, as believed before. However, conformational flexibility in such cases was rarely probed, leaving the physical basis of such unusual association largely unexplained. It will be particularly interesting to know that how conformational flexibility in such cases has been modified to accommodate both an increase in stability and activity. Such information can potentially provide a deeper insight into the stability- flexibility-activity relationship in enzymes.

By performing multiple rounds of directed evolution and mutation recombination on a lipase “lipA” from mesophilic bacterium *Bacillus subtilis*, we created a very thermostable mutant named “6B” [14]. This mutant harbors 12 thermostabilizing mutations (A15S, F17S, A20E, N89Y, G111D, L114P, A132D, M134E, M137P, I157M, S163P and N166Y); contribution of each of the mutations in increasing stability has been estimated experimentally [15]–[17]. Melting temperature and thermodynamic stability of 6B is ∼78°C and ∼15.1 kcal/mol, which is ∼22°C and ∼3.7 kcal/mol higher than wild type enzyme [14]. Along with imposition of selection pressure for higher thermostability during directed evolution of the lipase, we constrained the evolutionary process by screening for mutants which did not compromise on activity at room temperature [14]–[17]. Consequently, 6B showed improvement in catalytic activity at room temperature, in addition to improved stability, measured against substrates; *para*-nitrophenyl acetate (PNPA) and *para*-nitrophenyl butyrate (PNPB). Comparative details of catalytic parameters of the two enzymes are given in table 1. Increase in 6B activity is comparable to the reported values of other enzymes with simultaneous improvement in stability and activity [7]–[13].

**Table 1:** Catalytic parameters of lipases at room temperature (∼20°C).

	WT	6B
K_m_ with PNPA (mM^−1^)	0.98±0.08	0.51±0.07
k_cat_ with PNPA (min^−1^)	220±15	414±17
K_m_ with PNPB (mM^−1^)	0.29±0.07	0.17±0.05
k_cat_ with PNPB (min^−1^)	261±28	462±37
Specific activity with PNPB (µM.min^−1^.mg of protein^−1^)	6	37

**Figure 1:**
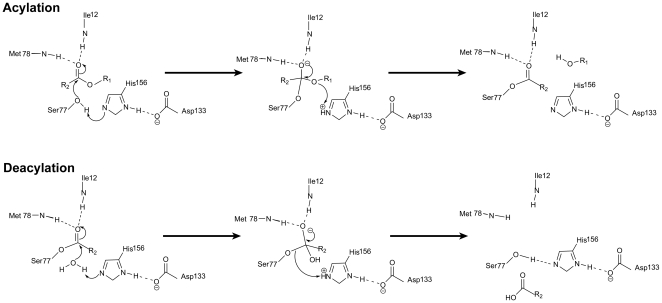
Catalytic mechanism of ester hydrolysis by *B. subtilis* lipase.

Pouderoyen *et al.* first solved the crystal structure of wild type *B. subtilis* lipase and identified the active site residues [18]. Substrate (ester) hydrolysis by *B. subtilis* lipase follows two steps reaction, acylation and deacylation (Fig. 1). The essential functional unit of *B. subtilis* lipase is the catalytic triad, which consists of S77, H156 and D133. Another important component of active center is oxyanion hole, constituted by peptidic NH groups of I12 and M78. As shown in figure 1, residues S77 and H156 are directly involved in catalytic reaction, acting as nucleophilic attacking group and general acid-base catalytic elements respectively. D133 acts as activator of H156 and helps in stabilization of positive charged developed on H156 during the course of reaction. Oxyanion hole stabilizes the negative charge developed onto the tetrahedral intermediates.

**Figure 2:**
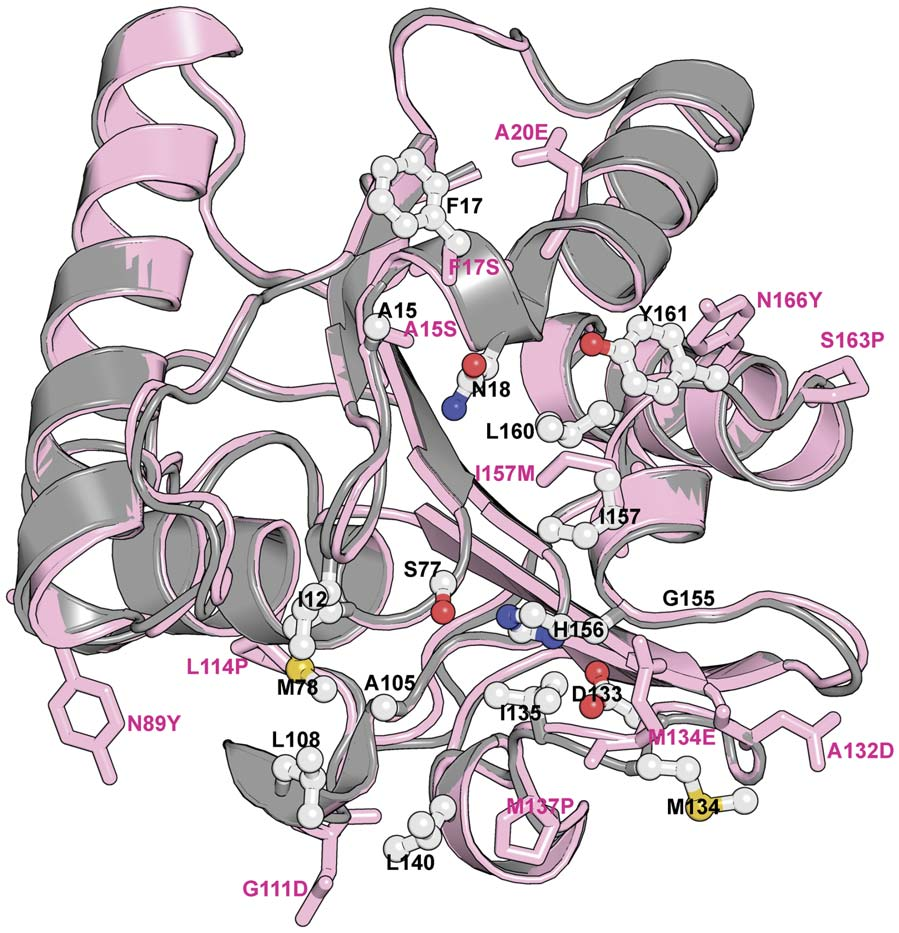
Positions of active site residues and mutations on lipase molecule. Wild-type (grey) and 6B (pink) crystal structures were overlapped. Side chain of active site residues are shown as ball and stick in elemental color (labeled with black) on wild type scaffold, while side chains of mutations are shown as stick in pink color (labeled with pink) on 6B scaffold. Active site residues: I12, A15, F17, N18, S77, M78, A105, L108, D133, M134, I135, L140, G155, H156, I157, L160 and Y161 [18]. Mutations: A15S, F17S, A20E, N89Y, G111D, L114P, A132D, M134E, M137P, I157M, S163P and N166Y. Stereo figure is shown in Fig. S1.

We have recently solved high-resolution crystal structure of 6B lipase and uncovered the structural basis of stabilization by individual mutations.[14]–[16] Eleven of the twelve mutations in 6B are involved in better anchoring of loops to rest of the protein molecule or increasing their rigidity through Xaa→Pro (Xaa = any amino acid) mutations. Notably, many of the mutations are either on the active site residues (A15S, F17S, M134E and I157M) or very close to them (Fig. 2 and S1). Most significantly, three of the stabilizing mutations namely A132D, M134E and I157M are adjacent to two of the catalytic triad residues (D133 and H156). Hence, it is reasonable to assume that stabilization through mutations might have rigidified the active site of 6B lipase. In the present study, we have used molecular dynamic (MD) simulation and time-resolved fluorescence anisotropy decay to establish that active site of 6B lipase is indeed more rigid than wild type. We further investigated the possible origin of high activity of 6B from its rigid active site.

## Results

### Molecular Dynamic Simulation

**Figure 3:**
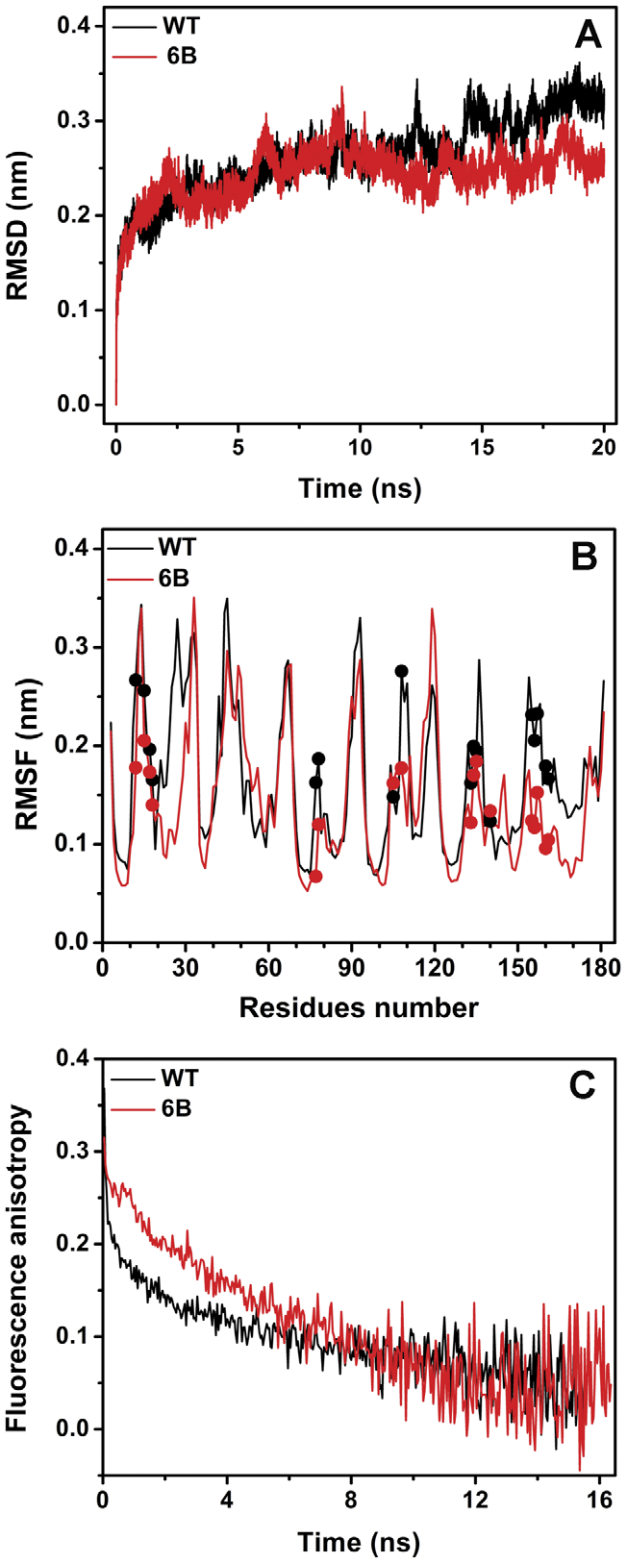
Active site dynamics of wild type and 6B lipase. (A) RMSD of C_α_ atoms of wild type and 6B lipases from their energy minimized crystal structures as a function of MD simulation time. For clarity, single simulation data is shown for both wild type and 6B lipase while others are presented in Fig. S2A. (B) RMSF of C_α_ atoms of individual residues during 2–20 ns simulation time. Active site residues positions are shown as solid spheres. (C) Typical time-resolved fluorescence anisotropic decay profiles of acrylodan attached to C77 in wild type and 6B lipase background.

Molecular dynamic simulation is a well-established method to computationally probe the structure and dynamics of biological macromolecules. This method has earlier been used to establish the relationship of protein dynamics to stability and enzyme activity [2], [19]. We performed three 20 ns molecular dynamics simulation of both wild type and 6B lipase at 293 K using GROMACS [20] by standard protocol followed by data analysis by same. 293 K (20°C) has been opted as the simulation temperature for the relevant comparison to the enzyme activity at room temperature. Figure 3A shows the root mean square deviation (RMSD) of C_α_ atoms of two protein structures as a function of simulation run time in reference to their respective energy minimized crystal structures. RMSD of both the proteins in all the simulations stabilizes very soon (<1 ns). To probe the flexibility of two molecules, data from all the three simulations were combined and the root mean square fluctuation (RMSF) for C_α_ atoms for all residues were compared for 2–20 ns MD runs (Fig. 3B). Higher value of RMSF means higher flexibility. As obvious from figure, barring few residues, RMSF of most of the 6B residues including active-site ones are lower than wild type protein that corroborates with the overall more rigid structure of 6B molecule (including active-site) than wild type protein. We obtained similar results while RMSF of all residue atoms are taken into consideration (Fig. S2B). Evidently, MD simulation suggests that active site of 6B lipase is indeed more rigid than wild type enzyme.

### Time-resolved fluorescence anisotropy

We further probed the active site dynamics of wild type and 6B lipases by time-resolved fluorescence anisotropy measurement. This method has been widely used to probe the flexibility of macromolecules [21]–[23]. It requires a fluorescent probe at specific site, whose flexibility is under investigation. Both wild type and 6B lipases harbor two tryptophan residues (W31 and W42), but they are spatially away from active site. Additionally, both the lipase variants are devoid of cystein residue. Hence, to probe active site dynamics using time-resolved fluorescence anisotropy, S77, catalytic serine, in both proteins was mutated to cysteine followed by specific conjugation with acrylodan, an extrinsic fluorophore [24]. More than 80% labeling by acrylodan was achieved. Choice of catalytic residue S77 for modification was appropriate, as during the course of catalytic reaction (ester hydrolysis) S77 forms covalent attachment with the fatty acid group of hydrolyzing ester (substrate), which is an intermediate state during catalysis (Fig. 1). Hence, covalently attached fluorophore at this site indeed represents the catalytic status of active site more closely than at any other position in active site. Neither mutation nor acrylodan labeling caused any structural change in lipases as judged by far UV circular dichorism (Fig. S3). Acrylodan labeled proteins was excited at 370 nm while emission was collected at 512 nm. Time-resolved fluorescence decay measurements were done using a high repetition rate picosecond laser (frequency doubled Ti-sapphire laser, Tsunami from Spectra-Physics Inc., USA) coupled to a time-correlated, single-photon counting (TCSPC) setup [25], [26]. Figure 3C shows typical time-resolved anisotropy decay profiles of acrylodan attached to wild type and 6B lipases. Both decay profiles could be fit satisfactorily as sum of two exponentials. Details of various parameters derived from anisotropic studies is given is table 2. Slower rotational correlation time (ф_slow_) belongs to the global tumbling of protein molecule, hence was similar for both proteins (∼8.9 ns). However, faster rotational correlation time (ф_fast_), representing sum of motional freedom of probe with respect to protein and segmental flexibility of local site (active site in present case), was different in two proteins. Its value is ∼0.21 ns in wild type while ∼4.05 ns in 6B. Lower value is due to faster depolarization which in turn represents higher flexibility of the local site. Furthermore, the amplitude associated with the faster correlation time is significantly smaller in 6B when compared to wild type indicating increased rigidity in 6B. Acrylodan anisotropic decay corroborates the MD simulation studies that active site of 6B lipase may be more rigid than wild type.

**Table 2:** Time-resolved fluorescence anisotropy decay.

	WT	6B
ф_fast_ (β_fast_)	0.21±0.02 (0.47)	4.04±0.04 (0.15)
ф_slow_ (β_slow_)	8.91±0.72 (0.53)	8.91±0.16 (0.85)
r_0_	0.31±0.01	0.29±0.00
r_ss_	0.12±0.01	0.21±0.00
χ^2^	1.05–1.16	0.98–1.09
τ_m_	3.75	2.87

ф_fast_ and ф_slow_ are fast and slow anisotropic decay rotational correlation times, while β_fast_ and β_slow_ are corresponding amplitudes. r_0_ is intrinsic (time zero) fluorescence anisotropy. r_ss_ is steady state anisotropy estimated from time-resolved fluorescence anisotropy decay experiments. τ_m_ is mean fluorescence lifetime obtained by magic angle measurements (Fig. S4 and Table S1). Both τ_m_ and ф are in ns. χ^2^ is a measure of goodness of fit. Closer the value to 1, better is fitting.

### Active site geometry during MD simulation

Our results from MD simulation and time-resolved fluorescence anisotropy decays suggest that active site of 6B lipase is more rigid than wild type lipase. This is in accordance with the prevailing idea of conformational stabilization being associated with increase in conformational rigidity. However, how a rigid active site can be capable of supporting higher enzyme activity? In any enzymatic reaction, even the simplest one substrate–one product ones, substrate goes through multiple geometric as well as electrostatic rearrangements during its chemical transformations. Correspondingly, for efficient catalysis, reorganization of enzyme active site is necessary so that it can change its shape to have complementarities with transforming chemical entity. Compelling need of dynamic fluctuations or flexibility for enzyme catalysis often originates from the simple fact that it bestows enzymes the capability of active site reorganization and allows it change its conformation during catalysis. However, relative level of active site reorganization during enzyme catalysis was shown to vary depending upon enzymes [27]–[29].

**Figure 4:**
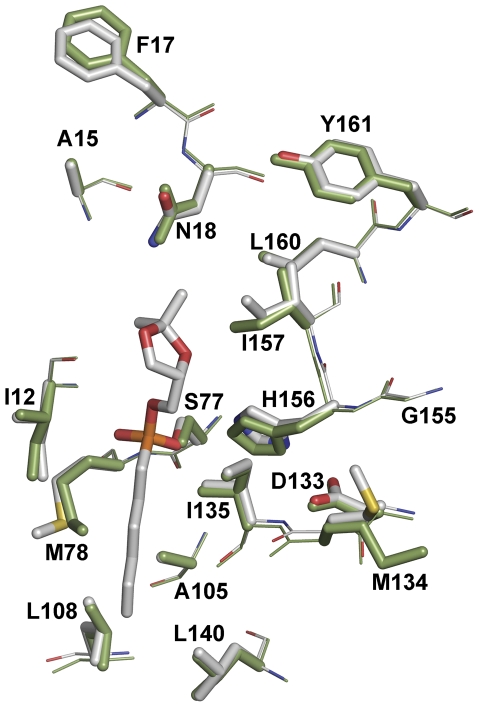
Active site in transition state bound and free form. Structural overlap of active site of the free wild type lipase and in complex with covalently attached transition state analog (chain A of PDB id: 1I6W and 1R4Z). Transition state analog is O-[(R)-1,2-O-isopropylidene-sn-glycerol]6-hexenyl phosphonate [34]. Free enzyme is shown in green while complex is shown in elemental color. Side chains are shown as sticks while backbone as lines. Stereo figure is shown in Fig. S6.

**Figure 5:**
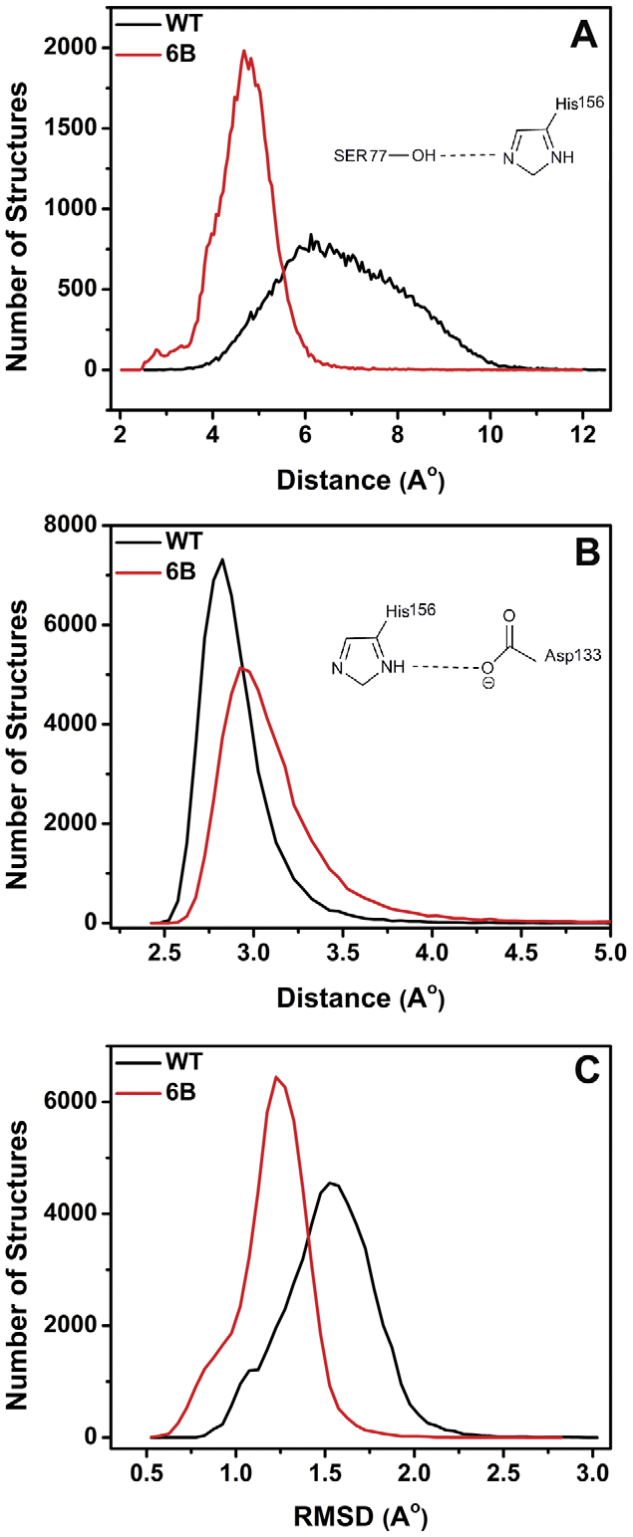
Active site geometry of wild type and 6B lipase during 2–20 ns MD simulations. (A) Frequency distribution of MD simulation structural snapshots as a function of distances between hydroxyl oxygen of S77 and imidazole nitrogen of H156. (B) Frequency distribution of MD simulation structural snapshots as a function of distances between imidazole nitrogen of H156 and carboxylate oxygen of D133. (C) Frequency distribution of MD simulation structural snapshots as a function of RMSD of their catalytically important atoms (hydroxyl oxygen of S77, imidazole nitrogens of H156, carboxylate oxygen of D133 and peptidic nitrogens of I12 and M78) to that of transition state analog bound crystal structure (PDB id: 1R4Z, Chain A).

As evident from Figure 1, ester substrate undergoes several structural changes during catalysis by lipases and esterases. However, recent work by Smith *et al.* [30] on serine esterases (including *B. subtilis* lipase), using the quantum mechanical and molecular mechanical techniques, has shown that these enzymes require minimal reorganization in active site during catalysis. The active sites in these enzymes were found to have pre-organized geometry that largely minimizes conformational reorganization during catalysis. We compared the crystal structures of wild type *B. sublilis* lipase in free form and in complex with a phosphonate inhibitor, an analog of 1^st^ tetrahedral transition state [31]. Overlap of the two structures showed no major difference in the position of any active site residues (Fig. 4 and S6), suggesting that during catalysis the overall positions are fixed (least movement) as earlier put forward by Smith *et al.* [30] It also suggest that active site crystal structure of apo (free) form of lipase is the catalytically competent structure, capable of carrying out the reactions related to catalysis without undergoing much structural change. These investigations imply that enhanced activity of 6B may originate from reduction in non-productive fluctuations that keep the active site of wild type lipase away from optimal geometry for catalysis. To examine this possibility, information from molecular dynamics were employed. Structural coordinates from all the simulations with the time interval of 1 ps were used. This resulted in a total of 60,003 structural snapshots for each lipase variant. We probed the change in active site geometry in the MD structures between 2–20 ns simulation time (54,003 structural snapshots) in comparison to transition state analog bound crystal structure using two important geometrical parameters; (i) hydrogen bonding setup of catalytic triad and (ii) overall geometry of catalytically important atoms (hydroxyl oxygen of S77, imidazole nitrogens of H156, carboxylate oxygen of D133 and peptidic nitrogens of I12 and M78). As shown in Fig. 5A, distance between hydroxyl oxygen of S77 and imidazole nitrogen of H156 was lower in large number of MD structures of 6B than wild type lipase. Average value of this distance was 6.8±1.1 Å and 4.7±0.6 Å during MD simulations of wild type and 6B lipase respectively. This distance was 2.7 Å in transition state bound crystal structure. However, distance variation between the carboxylate oxygen of D133 and imidazole nitrogen of H156 was relatively overlapping in the two simulations (Fig. 5B). Average value was 2.9±0.1 Å and 3.0±0.2 Å in case of wild type and 6B lipase respectively, which is in close agreement with the transition state bound crystal structure (3.1 Å). This suggests that S77-H156 hydrogen bond was relatively more stable in 6B than in wild type lipase while H156-D133 hydrogen bond was equally invariant in both cases. Clearly, catalytic triad setup was more preserved in 6B lipase than in wild type. Fig. 5C shows the RMSD values of catalytically relevant atoms geometry (defined before) amongst these MD snapshots in reference to transition state analog bound crystal structure. As obvious, larger fraction of structures during MD simulation preserved the geometry closer to the catalytic conformation in 6B than wild type lipase. We found similar result when wild type and 6B MD simulation structures were compared in reference to their respective crystal structure (Fig. S7). Both the active site geometric parameters suggest that conformational sampling in 6B by dynamics or thermal fluctuation is indeed overall much closer to catalytically competent conformation(s) than in wild type lipase. In other words 6B preserves it catalytically competent active site geometry better than wild type lipase as an effect of increase in its active site rigidity.

## Discussion

Numerous studies have attempted to uncover the possible relationship of conformational dynamics to enzyme activity. Various techniques such as NMR, MD simulation and single-molecule fluorescence were used to probe dynamics of many enzymes like cyclophilin A, adenylate kinase, peptidylprolyl isomerase catalytic domain, dihydrofolate reductase and flavin reductase [32]–[38]. These studies implicated that conformational dynamics significantly contributes to the catalytic power of enzymes. However, the exact mechanistic details on how dynamics contribute to enzyme catalysis are unclear. Other unresolved and challenging issue is the quantitative measure of influence of dynamics on enzyme activity. Mutations that perturb dynamics have been found to differ largely on their influence on enzyme activity. While mutations R55A, R55K and F113W decreased the activity of cycophilin A to <1%, H70A and K82A mutants of the same enzyme retained the activity comparable to wild type despite the fact that all the mutations perturbed the enzyme dynamics [32]. Similarly, while both mutations N23PP and S148A supposedly knocked out dynamics in dihydrofolate reductase, they influenced enzyme activity to different extent; former decreased the activity by ∼16 fold but later was almost as catalytically active as wild type [38]. In recent past, it has also been strongly emphasized that role of conformational dynamics in enzyme catalysis is negligible [39]–[41].

Despite of conceptual ambiguity and contradictory reports on role of dynamics in enzyme catalysis, prevailing notion is for efficient catalysis enzymes need to acquire structures that are compatible with binding to cofactor, substrate and its various forms while undergoing chemical transformation in the active site. And conformational dynamics or flexibility plays the vital role of a lubricant helping enzymes to do so. This implies that decreasing flexibility or increasing rigidity decreases the enzyme activity, as shown previously by many studies [29], [42], [43] and derived from comparison of mesophilic-thermophilic homologous enzyme pairs [1], [3]–[5]. In contrast, present study on *B. subtilis* lipase suggest that increasing active site rigidity not necessarily decrease enzyme activity, rather as presented here, can be associated with increase in enzyme activity. It should be noted that in earlier reports of engineered enzymes which were both more stable and comparable/more active at room temperature than their parent molecule, most of the mutations were spread over regions of molecules which were away from active site [7]–[13]. This gives a possibility that in such enzymes native structure might be globally more rigid hence more stable than their parents, while flexibility of active site might be retained/improved resulting in comparable/increased activity. However, our study on *B. subtilis* lipase directly probed the dynamics of active site argues that stable version of lipase “6B” is not only structurally more stable and active than wild type protein, but its active site is also more rigid as postulated based on static crystal structures.

In conclusion, our study provides the evidence that positive correlation between conformational flexibility and enzyme activity need not be stringent but active site rigidity and enzyme activity can be simultaneously increased. Interestingly, Gutteridge and Thornton [28] have compared the crystal structures of sixty enzymes in free and in substrate (or analog) bound form. They concluded that most of the enzymes undergo minimal changes in active site structure during catalysis. In combination with suggestion made in present study, it can be implied that achieving higher activity through increasing active site rigidity is feasible in enzymes. This possibility has significant bearing in enzyme engineering. This can specifically help in rational design of enzymes that can be more stable than their parents without sacrificing activity by performing mutation near and in the active site, which is usually excluded in protein engineering [44], [45]. Additionally, active sites of proteins can be modulated through mutations for various other engineering purposes like alteration in ligand binding affinity and specificity etc. without losing the structural integrity or stability.

## Materials and Methods

### Protein expression, purification and concentration estimation

All the lipase variants were over-expressed and purified following reported methodologies [15], [16]. Protein concentrations were estimated using modified Lowry method [46]. Catalytic activity of lipases were estimated as described earlier [15], [16].

### Molecular dynamic simulations

MD simulations and analysis were performed using GROMACS simulation package [20] adopting GROMOS96 force field parameters. Crystal structures (PDB id: 1I6W for wild type and PDB id: 3QMM for 6B) were taken as starting point for simulation. Structures were solvated into a cubic box of SPC (single point charge) water molecules using periodic boundary. Energy minimization (EM) is done using steepest descent method followed by dynamics simulations of the whole system (protein and water) in the NVT ensemble at 293 K temperature with a time step of 2 fs. The electrostatic interactions were calculated using the particle mesh Ewald summation method [47] while constraints were applied on all bonds using the LINCS [48] algorithm. Each simulation took ∼25–30 days to complete. Root mean square deviation (RMSD) and root mean square fluctuation (RMSF) were calculated using g_rms and g_rmsf commands, which are part of GROMACS simulation package, respectively.

### Acrylodan labeling

50 µM (∼1 mg/ml) of S77C mutants of both wild type and 6B lipases was incubated with 250 µM of acrylodan (R_391_ in DMF = 20,000 M^−1^.cm^−1^) for >2 h in 50 mM sodium phosphate buffer (pH 7.2) at room temperature followed by extensive buffer exchange with same buffer in centrifugal filtering device (Amicon ultra-15, 10 K cutoff from Millipopre). Percentage acrylodan labeling was calculated by measuring concentration of acrylodan (R_372_ = 16,400 M^−1^.cm^−1^) and total protein. >80% acrylodan labeling was achieved.

### Time-resolved fluorescence measurements and analysis

Time-resolved fluorescence experiments were performed using a Ti-Sapphire picoseconds laser and time-correlated single-photon counting setup, coupled to a micro-channel plate photomultiplier tube as described earlier [25], [26]. For acrylodan fluorescence, 0.5 mg/ml protein samples in 50 mM sodium phosphate buffer pH 7.2 were excited at 370 nm at 20°C while emission is collected at 512 nm. 370 nm excitation radiations is generated using pulses of 1 ps duration of 740 nm radiation, frequency doubled to 370 nm by using a frequency doubler/tripler (GWU, Spectra Physics). For Tryptophan fluorescence, samples (1 mg/ml proteins in 50 mM sodium phosphate buffer pH 7.2) were excited at 295 nm (tripled output of 885 nm) and emission was measured at 337 nm (Text S1, Table S1and Fig. S5). The instrument response function (IRF) at 370 and 295 nm was obtained using a dilute colloidal suspension of dried nondairy coffee whitener. The width (full width at half maximum) of the IRF was ∼40 ps. For fluorescence lifetime measurements, emission data (∼10,000 peak counts) were collected by orienting emission polarizer at magic angle (54.7°) with respect to the excitation polarizer (Fig. S4 and S5A). For time-resolved fluorescence anisotropy measurements, the emission data were collected at 0° (*I*_II_) and 90° (*I*_⊥_).

Time-resolved fluorescence intensity decays were analyzed by de-convolution of observed sample decays with the IRF to achieve the intensity decay function represented by,
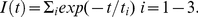
(1)Where, *I*(*t*) is the fluorescence intensity at time *t* and α*_i_* is the amplitude of the *i*th lifetime *τ_i_* such that Σα*_i_* = 1. The mean lifetime *τ_m_* = Σα*_i_τ_i_*.

Time-resolved anisotropy decays were analyzed by globally fitting *I*_II_ (*t*) and *I*_⊥_ (*t*) as
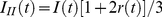
(2)

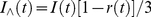
(3)Where, *I*_II_ (*t*) and *I*_⊥_(*t*) are fluorescence intensity when emission polarizer was oriented at 0° (*I*_II_) and 90° (*I*_⊥_) to the excitation beam. *r*(*t*), the anisotropic decay function was analyzed as a sum of two exponential terms,
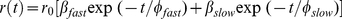
(4)Where, r_0_ is the intrinsic (time zero) fluorescence anisotropy. *φ*_fast_ and *φ*_slow_ are fast and slow rotational correlation times associated with amplitudes *β*_fast_ and *β*_slow_ respectively such that *β*_fast_+*β*_slow_ = 1.

## Supporting Information

Text S1 Information on time-resolved fluorescence intensity decay profiles of acrylodan and time-resolved fluorescence intensity as well as anisotropy decay of tryptophans.
(DOC)

Figure S1 Location of active site residues and mutations. Stereo figure for Fig. 2 (main text).
(TIF)

Figure S2 Active site dynamics by MD simulation. (**A**) RMSD of C_α_ atoms of wild type and 6B lipases from their energy minimized crystal structures in two simulations as a function of MD simulation time. (**B**) RMSF of all atoms of individual residue in 2–20 ns MD simulation time (all the three simulations). Spheres denote active site residues.
(TIF)

Figure S3 Far UC CD spectra of acrylodan labeled S77C mutant in wild type background and wild type lipase. Spectra were recorded in 0.1 cm pathlenght cuvette for 0.1 mg/ml proteins in 50 mM sodium phosphate buffer (pH 7.2) using a JASCO J-815 specropolarimeter. All reported spectra are an average of four accumulations. Wavelength scans were carried out in the Ellipticity mode at a scan speed of 50 nm/min, bandpass of 2 nm, at response time of 2 s and wavelength step of 0.5 nm. All spectra were corrected for buffer base line by subtracting the respective blank spectra recorded identically without the protein.
(TIF)

Figure S4 Typical time-resolved fluorescence intensity decay profiles of acrylodan attached to C77 in wild type and 6B lipase background.
(TIF)

Figure S5 Time-resolved fluorescence of tryptophans. Typical time-resolved fluorescence (**A**) intensity decay and (**B**) anisotropic decay profiles of tryptophans in wild type and 6B lipase.
(TIF)

Figure S6 Structural overlap of active site in free and transition state analog bound crystal structure of wild type lipase. Stereo figure for Fig. 4 (main text).
(TIF)

Figure S7 Active site geometry during MD simulation. Frequency distribution of RMSD of catalytically important atoms (hydroxyl oxygen of S77, imidazole nitrogens of H156, carboxylate oxygen of D133 and peptidic nitrogens of I12 and M78) between MD structural snapshots (2–20 ns) of wild type and 6B lipase and respective free enzyme crystal structures (PDB ids: 1I6W, Chain A for wild type and 3QMM, Chain A for 6B lipase).
(TIF)

Table S1 Parameters associated with time-resolved fluorescence measurements of acrylodan and tryptophans.
(DOC)
