# Supplementary material for: Role of Active Site Rigidity in Activity: MD Simulation and Fluorescence Study on a Lipase Mutant
Source: PLoS One. 2012 Apr 13;7(4):e35188. doi: 10.1371/journal.pone.0035188 (PMC3325981; doi:10.1371/journal.pone.0035188)
Supplement: Text S1 — Information on time-resolved fluorescence intensity decay profiles of acrylodan and time-resolved fluorescence intensity as well as anisotropy decay of tryptophans. (DOC) [file pone.0035188.s001.doc]

**Supplementary Information**

**Acrylodan fluorescence lifetime measurements:** Acrylodan was excited at 370 nm wavelength while emission is noted at 512 nm. Figure S4 shows typical fluorescence decay profiles of acrylodan fluorophore, attached to C77 in wild type and 6B lipase, used for lifetime measurements. Fluorescence decays in both proteins are best fitted with two lifetime components as judged by χ^2^ value. Various parameters obtained from fluorescence decays analysis were presented in table S1. Acylodan attached to both wild type and 6B lipase showed similar lifetime components (<2 ns and ~4 ns) but a difference in their fraction contribution to total fluorescence as can be seen in table S1, causing a change in mean lifetime, which is ~3.7 ns in wild type while ~2.9 ns in 6B.

**Tryptophan fluorescence lifetime and anisotropy measurements:** Lipase harbors two tryptophan residues at position 31 and 42, which are spatially away from active site as well as from each other. We also performed fluorescence lifetime and anisotropy decays measurements of tryptophans in both wild type and 6B lipase. 1mg/ml of proteins in 50 mM sodium phosphate buffer were excited at 295 nm (triple output of 885 nm) and emissions were recorded at 337 nm. Figure S5 shows the typical lifetime and anisotropic decay profiles of tryptophan residues from both lipases, while various fitting parameters are listed in table S1. Lifetime decay profiles in both proteins are best fitted with three components while anisotropic decays are best fitted with two components. Variation in the lifetimes and their corresponding amplitudes is subtle in two proteins giving a nearly equal mean lifetime ~1.3 ns. Also, both lipases showed similar anisotropic decay. In both proteins, larger of the two components representing global tumbling is ~8.5 ns while smaller component representing relatively local motions in the vicinity of tryptophans is ~3 ns. Hence, time-resolved tryptophan fluorescence anisotropic decays suggest that local environments of tryptophan residues are equally flexible. This is not unexpected as mutations are not as close to the tryptophan residues as are to active site. It is possible that although mutations brought rigidity to the active site, caused no such effect in tryptophan vicinity. It will be pertinent to mention that thermodynamic stability of most of the natural proteins is in the range of 5-15 kcal/mol. Thermodynamic stability of wild type lipase is ~11 kcal/mol, which is on the higher side of this stability spectrum. Hence, it will be safe to assume that wild type lipase is a very rigid structure and mutations in 6B could add very little to its global rigidity. Hence, time-resolved tryptophan anisotropic decay could not detect any increase in rigidity of 6B in comparison to wild type lipase.
