# Supplementary figures and images for: Role of Active Site Rigidity in Activity: MD Simulation and Fluorescence Study on a Lipase Mutant
Source: PLoS One. 2012 Apr 13;7(4):e35188. doi: 10.1371/journal.pone.0035188 (PMC3325981; doi:10.1371/journal.pone.0035188)

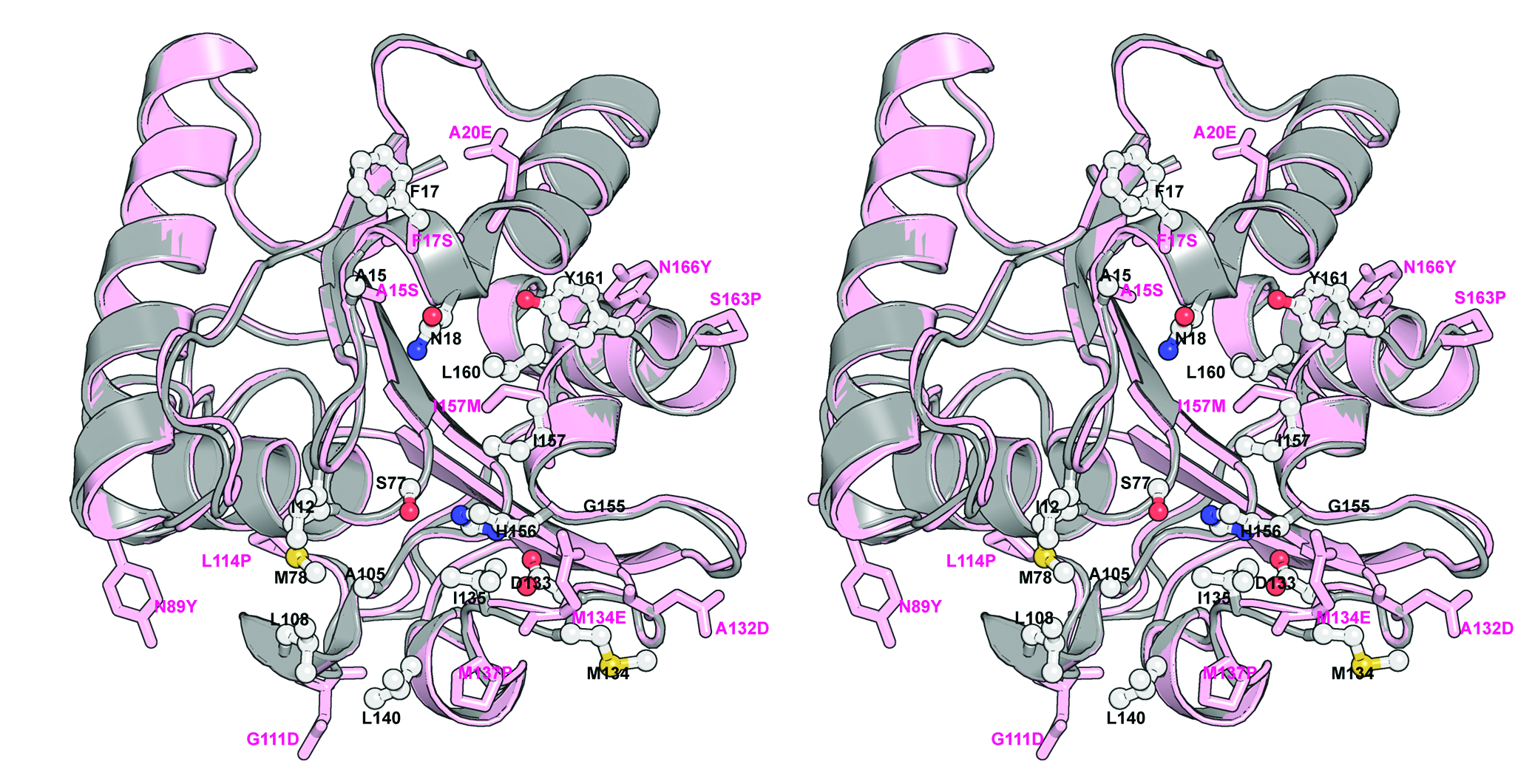

Supplement: Figure S1 — Location of active site residues and mutations. Stereo figure for Fig. 2 (main text). (TIF) [file pone.0035188.s002.tif]

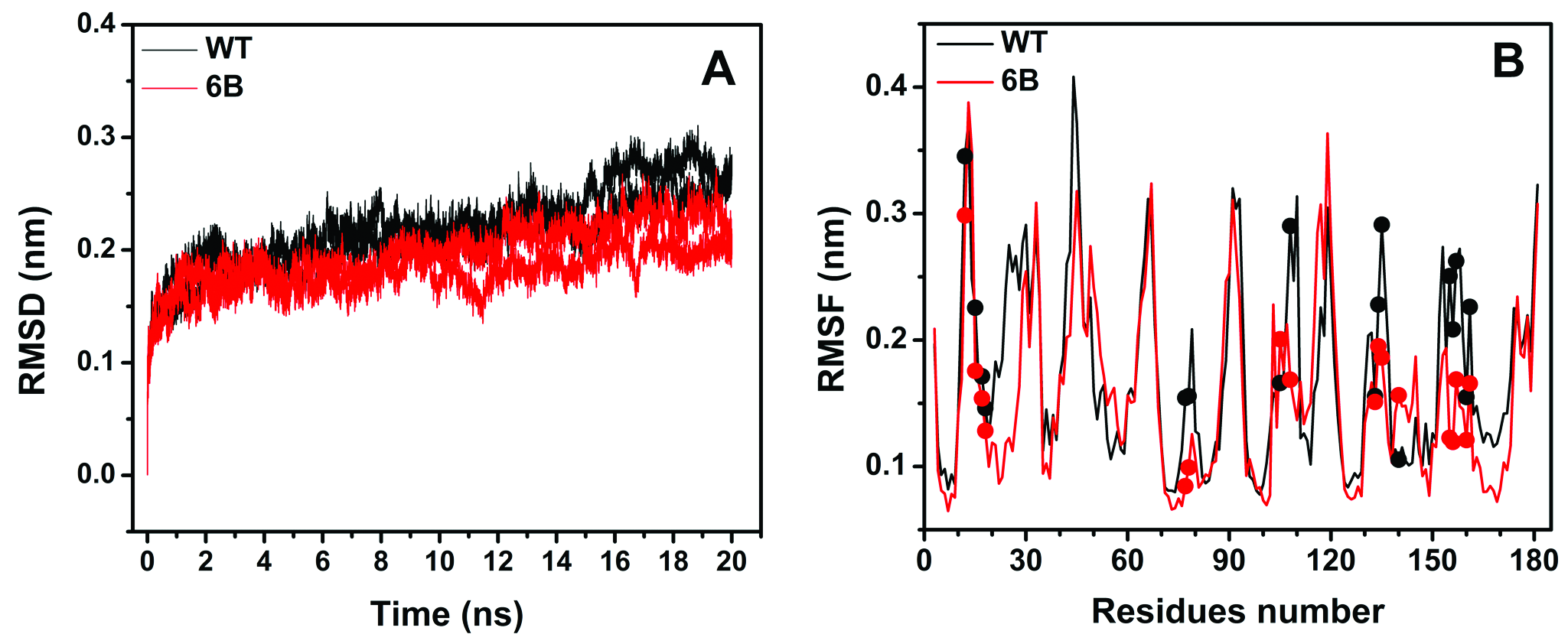

Supplement: Figure S2 — Active site dynamics by MD simulation. (A) RMSD of Cα atoms of wild type and 6B lipases from their energy minimized crystal structures in two simulations as a function of MD simulation time. (B) RMSF of all atoms of individual residue in 2–20 ns MD simulation time (all the three simulations). Spheres denote active site residues. (TIF) [file pone.0035188.s003.tif]

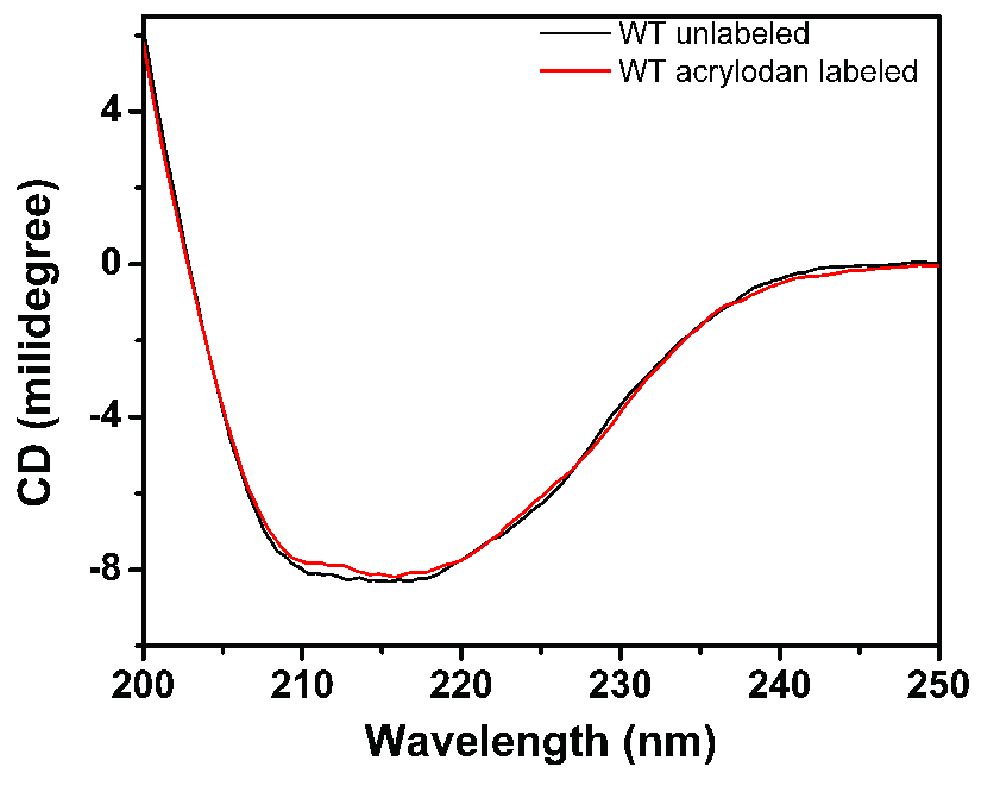

Supplement: Figure S3 — Far UC CD spectra of acrylodan labeled S77C mutant in wild type background and wild type lipase. Spectra were recorded in 0.1 cm pathlenght cuvette for 0.1 mg/ml proteins in 50 mM sodium phosphate buffer (pH 7.2) using a JASCO J-815 specropolarimeter. All reported spectra are an average of four accumulations. Wavelength scans were carried out in the Ellipticity mode at a scan speed of 50 nm/min, bandpass of 2 nm, at response time of 2 s and wavelength step of 0.5 nm. All spectra were corrected for buffer base line by subtracting the respective blank spectra recorded identically without the protein. (TIF) [file pone.0035188.s004.tif]

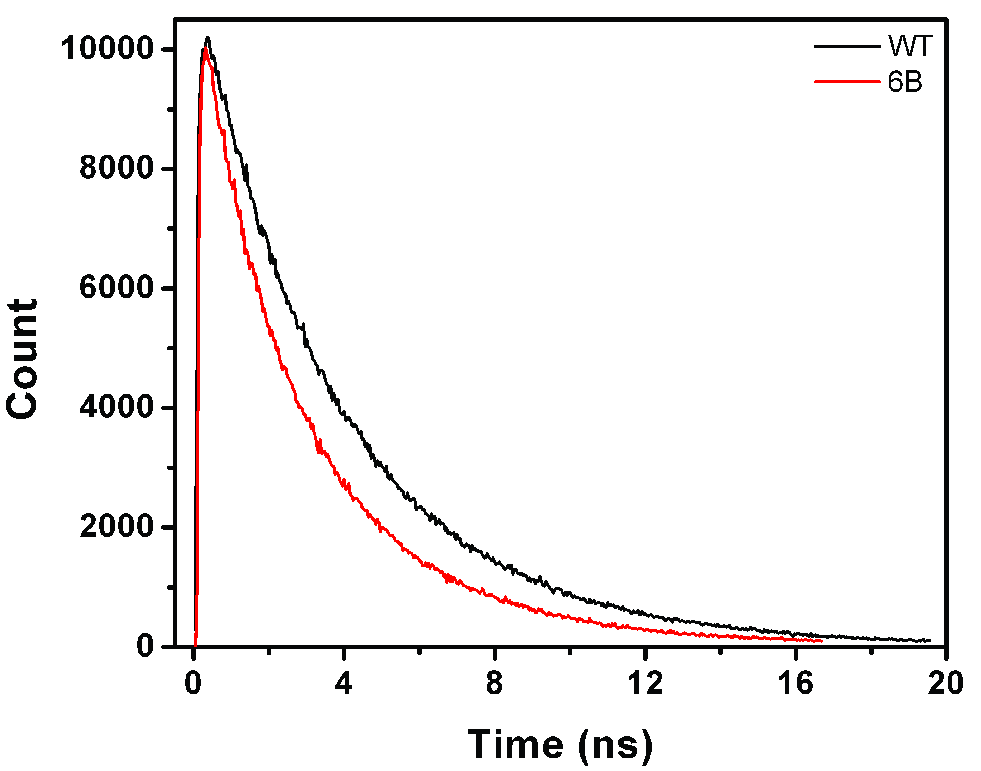

Supplement: Figure S4 — Typical time-resolved fluorescence intensity decay profiles of acrylodan attached to C77 in wild type and 6B lipase background. (TIF) [file pone.0035188.s005.tif]

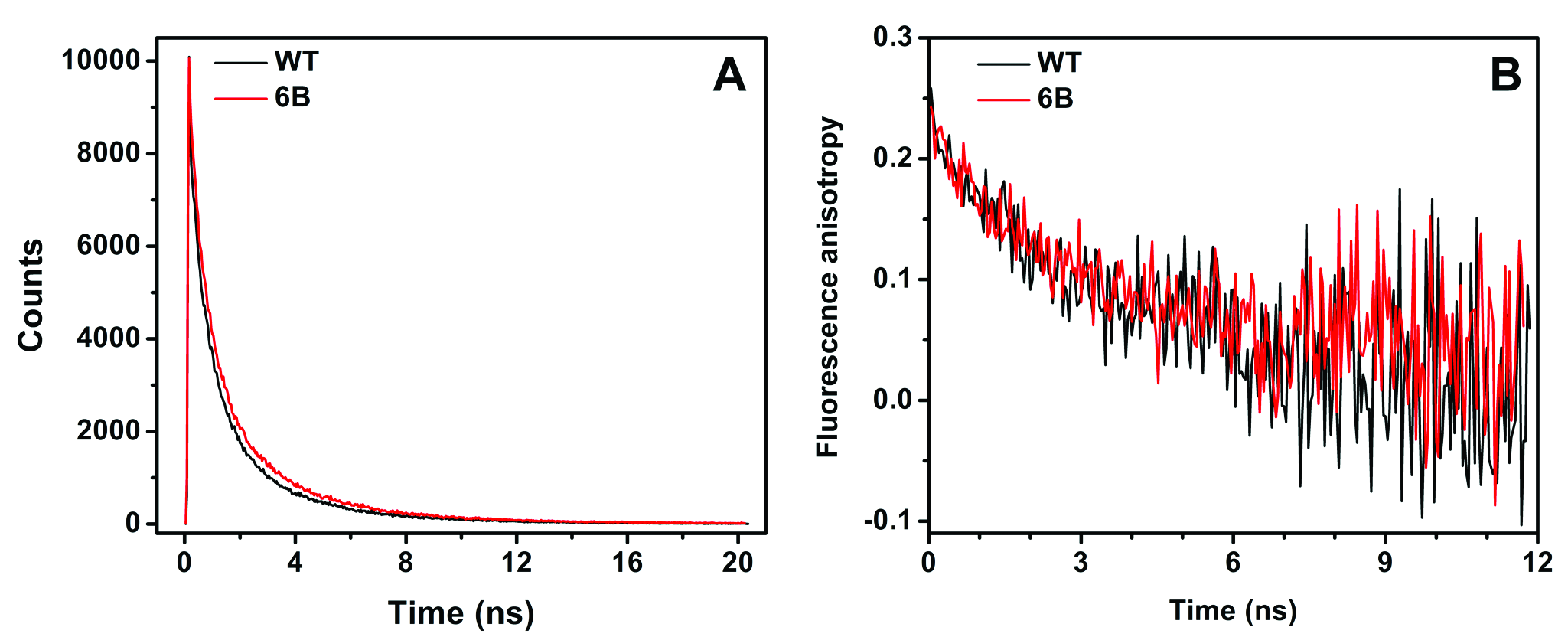

Supplement: Figure S5 — Time-resolved fluorescence of tryptophans. Typical time-resolved fluorescence (A) intensity decay and (B) anisotropic decay profiles of tryptophans in wild type and 6B lipase. (TIF) [file pone.0035188.s006.tif]

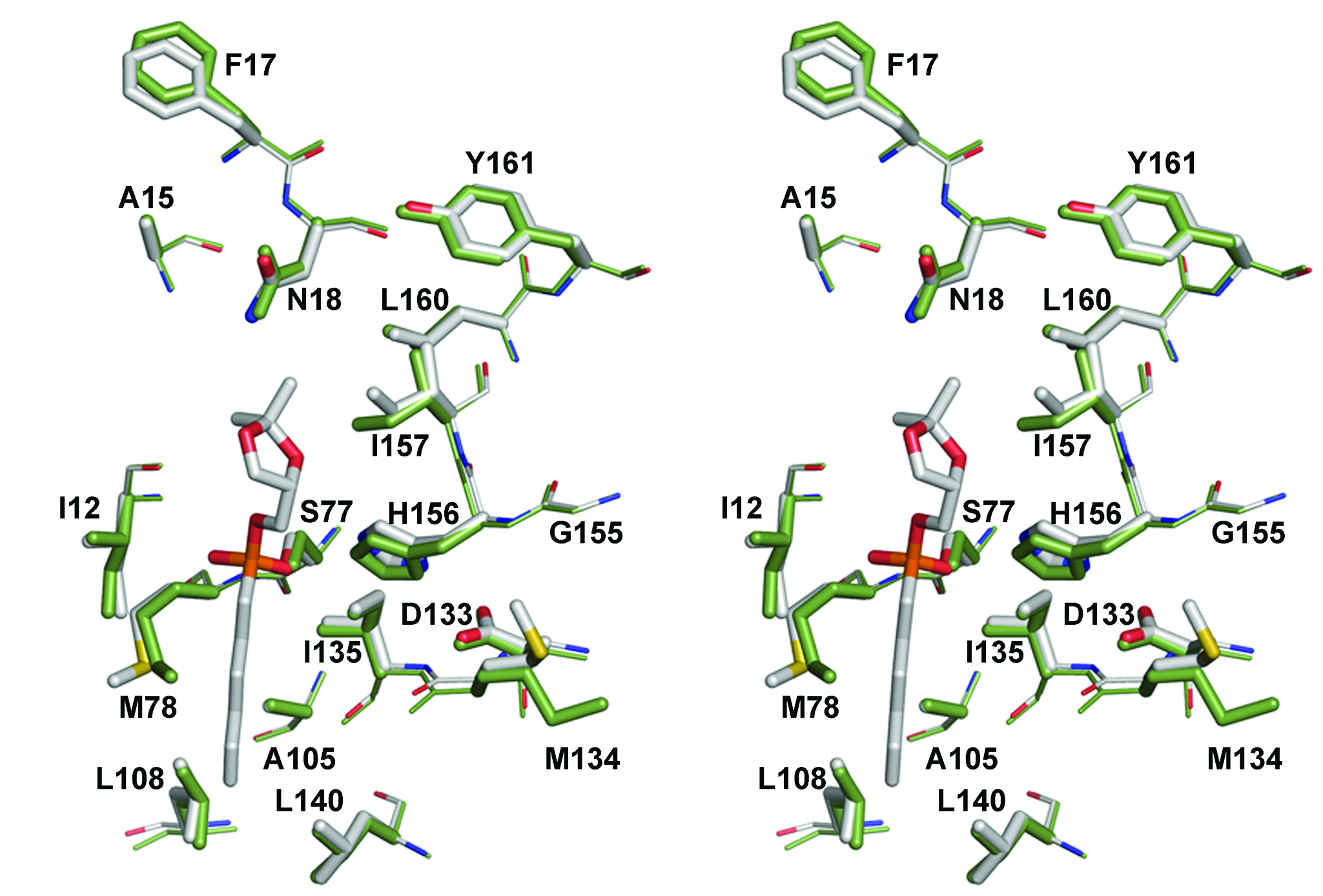

Supplement: Figure S6 — Structural overlap of active site in free and transition state analog bound crystal structure of wild type lipase. Stereo figure for Fig. 4 (main text). (TIF) [file pone.0035188.s007.tif]

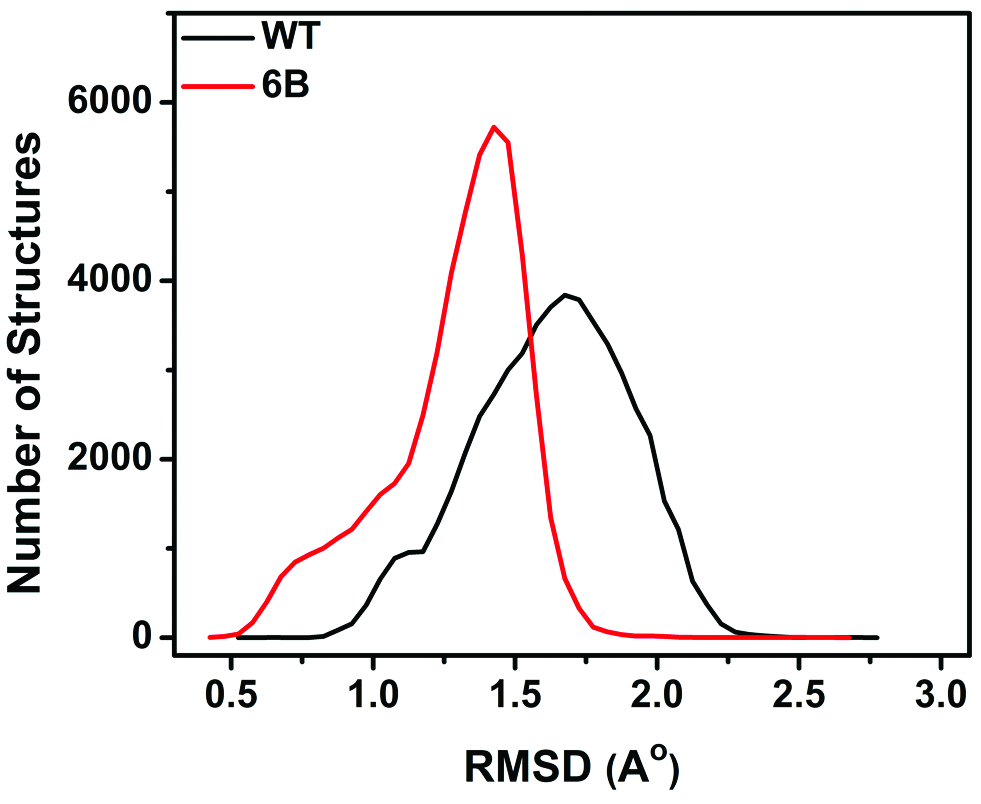

Supplement: Figure S7 — Active site geometry during MD simulation. Frequency distribution of RMSD of catalytically important atoms (hydroxyl oxygen of S77, imidazole nitrogens of H156, carboxylate oxygen of D133 and peptidic nitrogens of I12 and M78) between MD structural snapshots (2–20 ns) of wild type and 6B lipase and respective free enzyme crystal structures (PDB ids: 1I6W, Chain A for wild type and 3QMM, Chain A for 6B lipase). (TIF) [file pone.0035188.s008.tif]
