# Supplementary material for: Role of Active Site Rigidity in Activity: MD Simulation and Fluorescence Study on a Lipase Mutant
Source: PLoS One. 2012 Apr 13;7(4):e35188. doi: 10.1371/journal.pone.0035188 (PMC3325981; doi:10.1371/journal.pone.0035188)
Supplement: Table S1 — Parameters associated with time-resolved fluorescence measurements of acrylodan and tryptophans. (DOC) [file pone.0035188.s009.doc]

**Table S1:** Typical parameters associated with time-resolved fluorescence measurements

τ_i_ and α_i_ are fluorescence lifetimes and corresponding amplitudes. ф_fast_ and ф_slow_ are fast and slow anisotropic decay rotational correlation times, while β_fast_ and β_slow_ are corresponding amplitudes. r_0_ is intrinsic (time zero) fluorescence anisotropy. r_ss_ is steady state anisotropy estimated from time-resolved fluorescence anisotropy decay experiments. χ^2^ is a measure of goodness of fit. All fluorescence lifetimes (*τ_i_*) and rotational correlation times (ф_i_) are in ns. Closer the value to 1, better is fitting.

|  | **WT** | **6B** |
| --- | --- | --- |
| ***Acrylodan fluorescence lifetimes and their amplitudes*** | | |
| *τ_1_(*α*_1_)* | 1.63±0.23(0.13) | 1.87±0.05 (0.55) |
| *τ_2_(*α*_2_)* | 4.05±0.04(0.87) | 4.08±0.05 (0.45) |
| τ_m_ | 3.75 | 2.87 |
| χ^2^ | 1.03-1.19 | 1.00-1.08 |
| ***Tryptophan fluorescence lifetimes and their amplitudes*** | | |
| *τ_1_(*α*_1_)* | 0.58±0.01(0.54) | 0.56±0.05(0.56) |
| *τ_2_(*α*_2_)* | 1.49±0.01(0.30) | 1.79±0.08(0.32) |
| *τ_3_(*α*_3_)* | 3.40±0.04(0.16) | 4.21±0.02(0.12) |
| τ_m_ | 1.31 | 1.38 |
| χ^2^ | 1.04-1.11 | 0.99-1.11 |
| ***Tryptophan fluorescence anisotropy decay parameters*** | | |
| ф_fast_ (β_fast_) | 2.90±0.07(0.71) | 3.03±0.06(0.70) |
| ф_slow_ (β_slow_) | 8.50±0.10(0.29) | 8.5±0.10(0.30) |
| r_0_ | 0.22 | 0.21 |
| r_ss_ | 0.15 | 0.14 |
| χ^2^ | 1.53-1.60 | 1.48-1.64 |
